# Society Meetings

**Published:** 1915-02

**Authors:** 


### SOCIETY MEETINGS

---

Brief reports and announcements of meetings of societies of Western New York, and those of general scope, are requested from Secretaries. Copy should be on hand the fifteenth of the month. Full transactions will be published at cost of composition.

---

**DOCTOR: Is your Society properly represented here? If not, please bring our standing announcement to the attention of the members.**

---

**The Medical Society of the State of New York** will hold its hundred and ninth annual meeting in Buffalo, April 27-29. On account of the European War, this will probably be the largest medical meeting of the year, except perhaps that of the A. M. A. in San Francisco. Through the co-operation of the military authorities, the meeting will be held in the 65th Regiment Armory—not the old arsenal, now the City Convention Hall. This armory is one of the largest in the country and will afford accommodations for all activities of the meeting, except the annual banquet. A restaurant will be conducted in the building, there will be ample space for commercial and scientific exhibits, and an abundance of halls for general and section meetings. Even an automobile park will be provided on the armory grounds. No one need leave the building except to sleep, unless possibly to attend lectures to the laity which will be given by prominent visiting physicians and which will probably be held in the Masten Park High School across the street.

The choice of the armory is fortunate in another sense, as indicating the organization of the State Society as an arm of the state government. On the last night of the meeting, a regimental parade and review by Gen. Gorgas will be held.

We venture to assert that this meeting will be conducted to insure greater comfort and convenience to guests than any other gathering of the kind. There will be no waste of time in passing from one section to another, no mental strain in fixing one's attention on gall stones while, on the other side of a velvet (?) curtain, some one is discoursing on ventral fixation or an organization of "hundred-point" men is discussing the best methods of selling varnish.

The local committee of arrangements consists of the chairmen of the following sub-committees. Suggestions and offers of assistance will be gladly received. We understand that the annual meeting is entirely self-supporting, from the sale of concessions, so that no financial contributions will be asked.

Sub-Committees of Arrangements.

RECEPTION: Chas. G. Stockton, chairman, 436 Franklin Street; Arthur W. Hurd, Henry R. Hopkins, William H. Thornton, Henry C. Buswell, Herman E. Hayd, Edward J. Meyer, Harvey P. Gaylord, DeLancey Rochester, Allen A. Jones, Edgar R. McGuire, Thomas J. Walsh, Bernard Cohen, James A. Gardner, Francis E. Fronczak, Lee Masten Francis.

MEETING ROOMS: Nelson G. Russell, chairman, 469 Franklin Street; Albert H. Briggs, Renwick R. Ross, Stephen Y. Howell, Theodore M. Leonard, Arthur C. Schaefer.

PUBLICITY: A. L. Benedict, chairman, 228 Summer St.; William W. Quinton, George A. Himmelsbach.

LADIES: Edith R. Hatch, chairman, 2620 Main Street; Maude J. Frye, Myrtle A. Hoag, Lucy A. Kenner, Caroline Lichtenberg, Elizabeth Dort, Katherine Munhall.

TRANSPORTATION: Carl G. Leo-Wolf, chairman, 481 Franklin Street; William Gaertner, Robert E. DeCeu, Edward M. Tracy, Nelson W. Strohm.

BANQUETS AND HOTELS: Lesser Kauffman, chairman,

534 Elmwood Avenue; Joseph F. Whitwell, William G. Bissell, Earl P. Lothrop, Frederick J. Parmenter.

EXHIBITS AND AUDITS: Albert T. Lytle, chairman, 200 Lexington Avenue; Arthur G. Bennett, Julius Richter.

REGISTRATION AND INFORMATION: Edw. A. Sharp, chairman, 481 Franklin Street; John R. Gray, Clayton M. Brown, John L. Butsch, William L. Phillips, Frank N. Potts, Descum C. McKenney, Herman K. DeGroat, William F. Jacobs, Herbert A. Smith, William Ward Plummer, Augustus W. Hengerer, Nadina R. Kavinoky.

---

**The Medical Society of the County of Genesee** held its largest meeting January 7, at Batavia. Addresses were made by Dr. Grover W. Wende of Buffalo, President of the State Society; Dr. W. A. Howe of Phelps, former Deputy Commissioner of Health; Dr. Harvey J. Burkhart, State Dental Examiner and Mayor of Batavia, and Dr. C. V. Patchin of Dansville, Sanitary Supervisor.

---

**The Elmira Academy of Medicine** held its regular monthly meeting January 6. Dr. G. V. R. Merrill reported a case of Oophorectomy, diagnosis in question; Dr. Charles Haase showed X-Ray Photographs of Bone Disease.

---

**The Hospital Medical Society** of Rochester, met January 7. Dr. Bradford A. Richards presented a paper on Brain Abscess of Otitic Origin.

**The Rochester Academy of Medicine** held its annual meeting January 13. Dr. Joseph Roby gave a paper on The Medical Treatment of Hyperthyroidism and Basedow's Disease; Dr. Thomas Jameson on the Surgical Treatment of Hyperthyroidism and Dr. Curtis Jameson on The Administration of Anaesthetics in Operations on the Thyroid.

---

**The Rochester Pathological Society** met January 14. Dr. A. B. Wadsworth of Albany read a paper on Mechanism of Recovery in Diphtheria and in Pneumonia.

**The Buffalo Acadamy of Medicine** has held the following meetings since our last report:

Section of Surgery: January 6. Roentgenographic Examination of the Urinary Bladder, Dr. John Garratt; One Hundred Cases of Appendicitis, Dr. Thew Wright.

Section of Medicine: January 13. Traumatic Lesions of the Spinal Cord—Lantern Slides, Dr. Edward A. Sharp.

Section of Obstetrics and Gynaecology: January 20. Operative Treatment of Chronic Intestinal Stasis, Dr. Wm. S. Bainbridge, New York. Discussion by Dr. H. D. Meeker of N. Y., E. P. Lothrop and E. R. McGuire. Dr. Blaauw showed a case of epithelioma of the cornea.

